# A SAR Observation and Numerical Study on Ocean Surface Imprints of Atmospheric Vortex Streets

**DOI:** 10.3390/s8053321

**Published:** 2008-05-21

**Authors:** Xiaofeng Li, Weizhong Zheng, Cheng-Zhi Zou, William G. Pichel

**Affiliations:** 1 IMSG at NOAA/NESDIS E/RA3, WWBG, Room 102, 5200 Auth Road, Camp Springs, MD 20746-4304, USA; 2 NOAA/NCEP/EMC, 5200 Auth Road, Camp Spring, MD 20706, USA; 3 Center for Satellite Applications and Research, NOAA/NESDIS, Camp Springs, MD 20746, USA

**Keywords:** Remote Sensing, Ocean, SAR, RADARSAT, MODIS, AVS, NOGAPS

## Abstract

The sea surface imprints of Atmospheric Vortex Street (AVS) off Aleutian Volcanic Islands, Alaska were observed in two RADARSAT-1 Synthetic Aperture Radar (SAR) images separated by about 11 hours. In both images, three pairs of distinctive vortices shedding in the lee side of two volcanic mountains can be clearly seen. The length and width of the vortex street are about 60-70 km and 20 km, respectively. Although the AVS's in the two SAR images have similar shapes, the structure of vortices within the AVS is highly asymmetrical. The sea surface wind speed is estimated from the SAR images with wind direction input from Navy NOGAPS model. In this paper we present a complete MM5 model simulation of the observed AVS. The surface wind simulated from the MM5 model is in good agreement with SAR-derived wind. The vortex shedding rate calculated from the model run is about 1 hour and 50 minutes. Other basic characteristics of the AVS including propagation speed of the vortex, Strouhal and Reynolds numbers favorable for AVS generation are also derived. The wind associated with AVS modifies the cloud structure in the marine atmospheric boundary layer. The AVS cloud pattern is also observed on a MODIS visible band image taken between the two RADARSAT SAR images. An ENVISAT advance SAR image taken 4 hours after the second RADARSAT SAR image shows that the AVS has almost vanished.

## 1. Introduction

Atmospheric vortex street (AVS), resembling the classic Von Kármán vortex street in any fluid, can develop on the lee side of obstacles under the favorable wind conditions when wind flows over an inland or isolated topography. A typical AVS consists of a string of vortices with diameter of tens of kilometers and may persist from 100 to 400 km downstream of the obstacle. AVS plays an important role in modifying the horizontal and vertical structure of the wind, moisture and temperature near the island, and sometimes can cause safety concerns and affecting aviation operations. AVS is usually too small to be delineated by a synoptic observation network and too large to be observed by a single station [Chopra 1964; Etling 1989a]. Previous observations of AVS have been made by weather satellites since the 1960's. AVS's were detected in satellite cloud images [Walter and Fujita 1968; Thomson and Bowker 1977; Ferrier et al. 1996], but the relationship between cloud density and wind velocity is unclear [Pan and Smith 1999]. AVS also changes the low-level atmospheric wind pattern, and thus, has imprints on the sea surface. Li *et al.* [2000] and Young and Zawislak [2006] reported the observation of the sea surface imprints of such AVS imaged by synthetic aperture radar (SAR) image.

The analysis of this important marine atmospheric boundary layer (MABL) phenomenon has been primarily based on idealized laboratory experiments and conceptual theoretic models [e.g. Taneda 1965; Gaster 1969; Ericsson 1980; [Bibr b4-sensors-08-03321][Bibr b5-sensors-08-03321]; Sun and Chern 1994; Thomas and Auerbach 1994; Schär and Durran 1997; Burk et al. 2003]. Although conceptual models may provide a basic understanding on the structural development and the range of some parameters under ideal laboratory experiments with idealized topography, they have limitations in providing complete understandings on the natural three-dimensional patterns and time evolution of the AVS under actual atmospheric conditions with complex topography. In addition, since previous studies of AVS in the actual atmosphere are limited to only one or two satellite images, it is impossible to validate basic AVS characteristics derived from these idealized theoretical models. Here we present a case study of AVS off Aleutian Islands, Alaska using a RADARSAT-1 Synthetic Aperture Radar (SAR) image and the fifth-generation Pennsylvania State University (PSU)-National Center for Atmospheric Research (NCAR) Mesoscale Model [MM5, Grell et al. 1995] with actual atmospheric conditions. By comparing the AVS simulation with SAR images, we examine atmospheric vortex streets in the full time and space domains.

## 2. RADARSAT-1 Synthetic Aperture Radar observations

**Figure 1.**
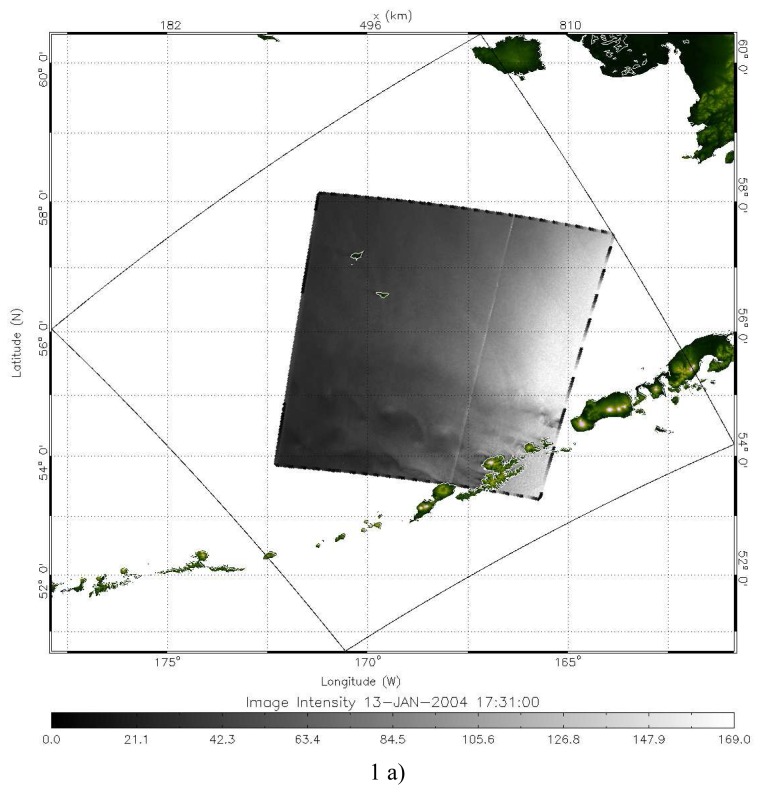

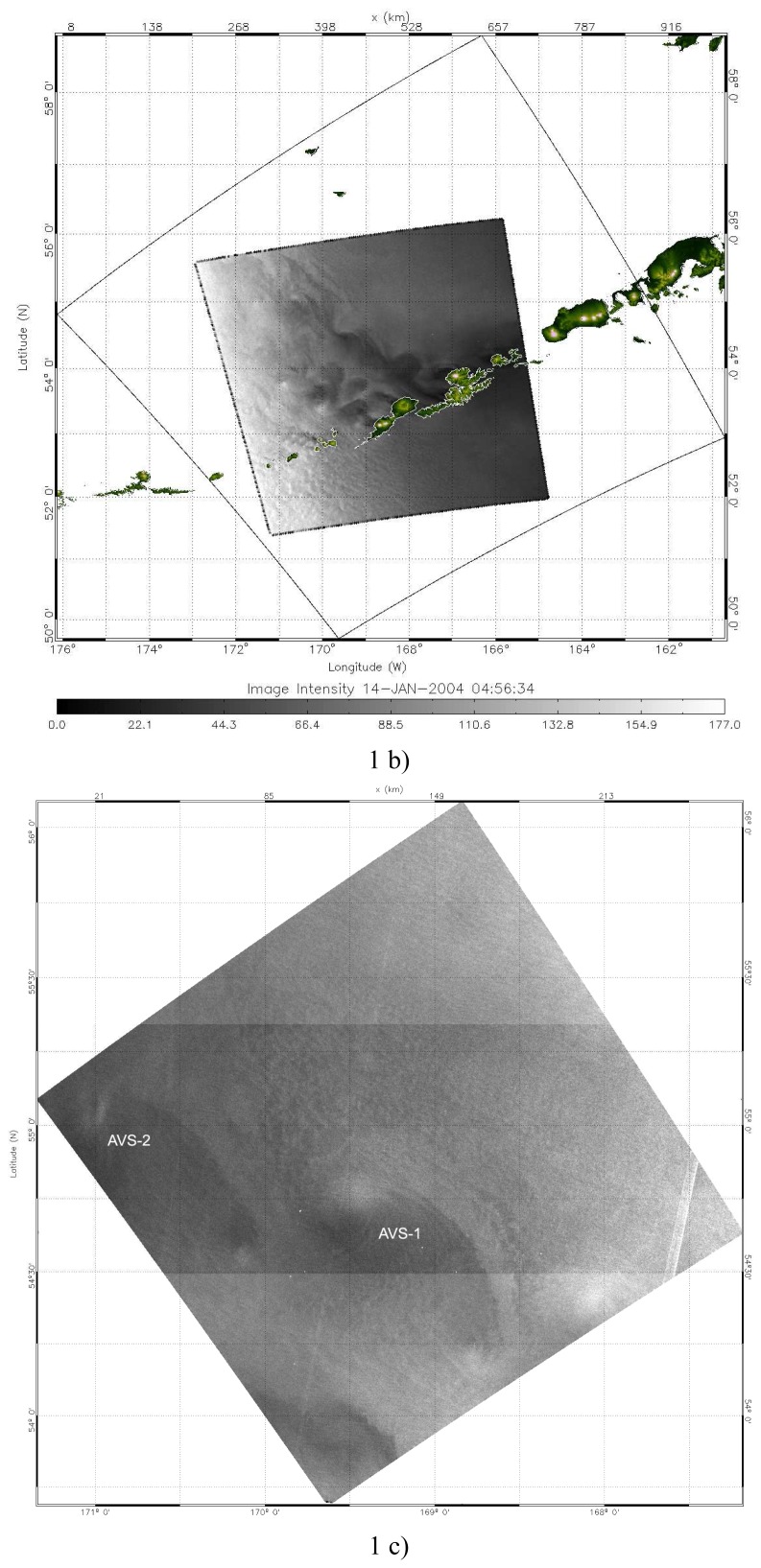
Two RADARSAT-1 ScanSAR wide images off a portion of the Aleutian Islands, Alaska. (a) 17:31:00 UTC on January 13, (b) 04:56:34 UTC on January 14, 2004. (c) An enlarged area on the lee side if Aleutian Island in Figure 1a showing the detailed AVS structure.

Two RADARSAT-1 SAR images shown in Figure1a and 1b were extracted from RADARSAT-1 ScanSAR Wide B scenes that were processed at the Alaska Satellite Facility (ASF). The RADARSAT-1 SAR operates in C band with HH polarization. The images used in this study have a spatial resolution of 100 m with a pixel spacing of 50 m. The image swath for the ScanSAR wide mode is about 450 km. The first image covers the central part of the Aleutian Islands, Alaska and is centered at roughly 55.7°N, 168.0°W and was acquired at 17:31:00 UTC on January 13, 2004. The dark pattern in the middle of the AVS represents low backscatter from a smoother sea surface associated with lower wind speed in the island's lee shadow region. The bright spots represent strong wind regions. Well-defined AVS can be clearly seen downwind of Makushin Volcano in Unalaska Island. There are 3 pairs of vortex shedding on this vortex street. In addition, vortex trails also formed in lee of the Umnak Island but the image covered only portion of them. Part of Figure 1 is enlarged and given in Figure 1c to highlight the distinguished AVS structures.

The SAR image depicted in Figure 1b is shifted slightly to the south, which is centered at about 54.0°N, 168.5°W and was acquired at 04:56:34 UTC on January 14, 2004, about 11 hours after the first SAR image (Figure 1a) was taken. Three pairs of distinctive vortices shedding in the lee of Mount of Recheshnoi and Mount Vsevidof, Alaska were also observed. The distance between two vortices in the downstream direction was about 64 km and 73 km, respectively. This vortex street showed irregular, with an unsymmetrical structure with well-structured vortices on the left side but poorly formed vortices on the right side. Oscillatory-type wake patterns are observed downwind of the Unalaska Island and the Tulik Volcano of Umnak Island.

**Figure 2.**
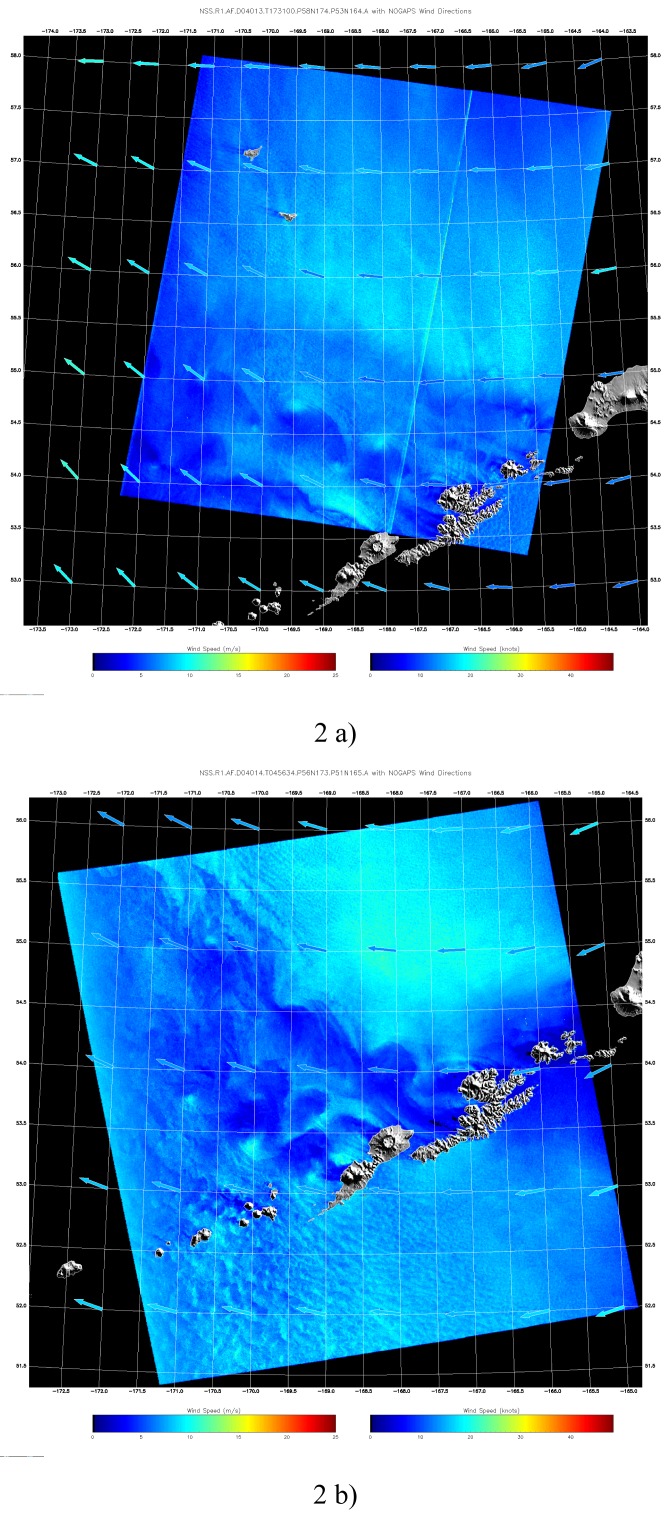
The wind speed extracted from the calibrated SAR image by using a CMOD5 model modified for HH polarization. Wind directions are obtained from Navy's NOGAPS model. (a) SAR wind image from Figure 1a; (b) SAR wind image from Figure 1b.

SAR measures the variation in normalized radar cross section (NRCS) from the wind-roughed ocean surface. NRCS is a function of both wind velocity and direction. Since SAR has only one azimuth viewing angle, we can not get the wind direction directly from the SAR measurement. To derive the wind velocity, one must obtain the wind direction independently from another source [Li et al. 2004a; Monaldo, et al., 2004], i.e., operational meteorological model output, wind-aligned features in the SAR image with 180-degree ambiguity, or coincident scatterometer measurements. In this study, the wind images were derived using the CMOD5 wind retrieval model modified for HH polarization (Figure 2a and 2b). The wind direction is obtained from the gridded Navy NOGAPS operational model matched closet in time with the SAR observation. The color of the wind arrows represents the wind speed from the NOGAPS model. One can see that the SAR derived wind speed closely matches the wind from the NOGAPS model (similar color).

The SAR-derived wind image shows the vortices pattern on the lee side of the mountain. The wind speed has a sharp gradient across the vortex boundary, which changes from 17 m s^-1^ to 2 m s^-1^ within the vortex. In the next section, we will show that the SAR-derived sea surface wind pattern and amplitude of the speed are in good agreement with the actual model simulation results. The limitation of the SAR wind retrieval in this study is that the NOGAPS products have a spatial resolution of 1 by 1 degree, so that its wind direction remains uniform within the small vortex. As a result, we are unable to evaluate the small-scale wind variations within the vortices.

## 3. Mesoscale Meteorological Model Description

The PSU/NCAR MM5 is used to simulate the AVS and investigate its detailed structure and evolution. MM5 is a limited-area, non-hydrostatic, terrain-following sigma-coordinate model designed to simulate or predict mesoscale atmospheric circulation [Grell *et al.* 1995]. The MM5 model can simulate the low-level atmospheric wind in about the same resolution as measured by SAR instrument. Our recent study shows that the model simulated wind pattern is closely resembled to the SAR observation [Li *et al.*, 2007].

We use a two-way interactive, triply nested-grid technique with a uniform horizontal grid resolution of 9, 3 and 1 km for each computational domain, respectively. The coarse domain, centered at 53.0°N and 167.5°W, uses 181 by 175 grid points, the medium domain uses 175 by 157 grid points, and the finest domain uses 334 by 247 grid points, which covers the area around 52.6°-54.9°N and 171.4°-166.3°W. A total of 32 full σ levels in the vertical are used with the model top at 100 hPa and stretched to give cells of height near 10 m close to the surface. The primary model physical settings are the same as these in our previous studies, i.e. [Li et al. 2004b and Li et al. 2007]. The outermost coarse-mesh lateral boundary conditions are specified by linearly interpolating the National Centers for Environment Prediction (NCEP) 6-hourly Final Analyses (FNL) at a resolution of 1° × 1° degree. The sea-surface temperature is updated everyday according to the FNL data. The model is initialized for both coarse and medium domains at 0000 UTC 12 January 2004 with FNL data. The finer domain is initialized after 24-h model integration and then integrates continuously to 1200 UTC 14 January 2004.

**Figure 3.**
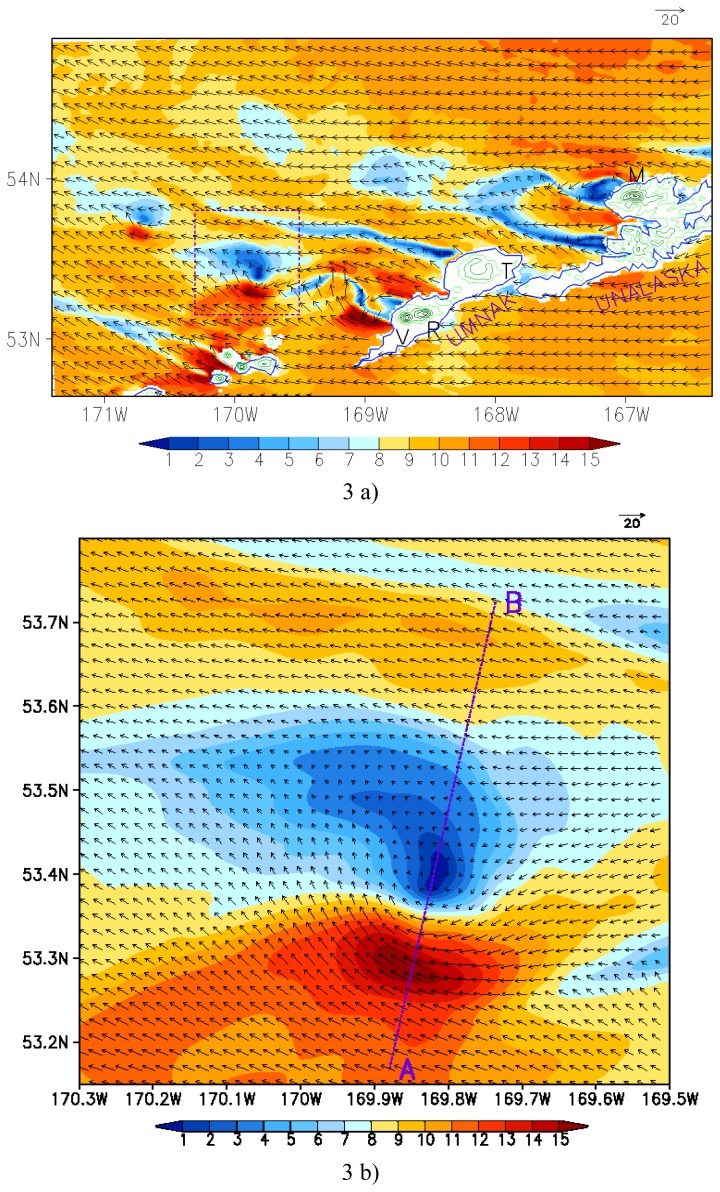
MM5 model simulation of the AVS at 0500 UTC on January 14, 2004. (a) Wind fields (in m s^-1^) at the lowest model level (σ = 0.9986, i.e. about 10 m above the sea surface). Black arrows represent wind vectors and color-coding denotes wind speeds. Green lines denote topography over the islands with a contour interval of 300 m. The letters “M”, “V”, “R” and “T” represent Markushin Volcano, Mount Vsevidof, Mount Recheshnoi and Tulik Volcano, respectively. The rectangle domain is the lateral boundaries for figure b; (b) Detailed vortex structure within the rectangle domain in figure a. The line AB is the width of AVS.

The digital elevation of 30 seconds United States Geological Survey (USGS) topography data is used in the simulation. In the 1-km model finest resolution, Mount of Recheshnoi and Mount Vsevidof is visible with a model height of 1500 m, and Makushin Volcano in Unalaska Island is visible with a model height of 1800 m (Figure 3a).

## 4. Numerical Simulation of Atmospheric Vortex Streets

The 10-m wind field above the sea surface simulated by the model for 0500 UTC 14 January 2004, about 3 minutes after the second SAR data acquisition, is presented in Figure 3a. It can be seen that the model reasonably reproduces the shadow patterns (i.e. areas of low wind speed) seen in the SAR image downwind of mountain. Oscillatory-type wake patterns can be seen downwind of Unalaska Island and Tulik Volcano of Umnak Island. In the lee of Mount of Recheshnoi and Mount Vsevidof of Umnak Island, a well-defined vortex street is generated with a similar asymmetrical structure to that in the SAR image. The distance between vortices downwind is about 55 and 70 km, respectively, close to those estimated from the SAR image. Moreover, the vortex street is not exact straight, and the span wise separation does not show constant either. As mentioned earlier, the laboratory or conceptual theoretic models provide a basic understanding of the AVS structure and evolution [Chopra and Hubert 1964; Etling 1989a, Li, et al., 2000]. However, in real atmospheric and topography conditions this phenomenon exhibits complexity that is difficult to be described with the laboratory or conceptual theoretic models. Our observation and simulation here show that the AVS pattern is quite different from the same type of feature generated behind circular cylinder in laboratory or conceptual theoretic models [i.e. Taneda 1965; Schär and Durran 1997].

To clarify details of the structure of the above three vortices, we examine the vortex located within the rectangle domain in Figure 3a. The enlarged surface wind field within this domain is shown in Figure 3b. Outside the vortex along the left of the AVS, the strong wind can reach up to 15 m s^-1^, while within the vortex the lower wind speed is only near 1 m s^-1^. Moreover, a small-scale vortex is simulated in the weak wind region and its horizontal scale is about 20 km, which is similar to that in the SAR image. The model-simulated vortex scale and the strong or weak wind region agree well with the SAR-wind image. A strong horizontal wind shear occurs in the south of this small scale vortex. In the downwind direction, the wind is stronger on the left-hand side but weaker on the right-hand side. Therefore, the wind is not strong enough to be defined as vortices. The background air flow is about 8-9 m s^-1^, but within the vortex street the wind is fairly weak at only about 1 m s^-1^. Therefore, the vortex is located in a low energy zone.

**Figure 4.**
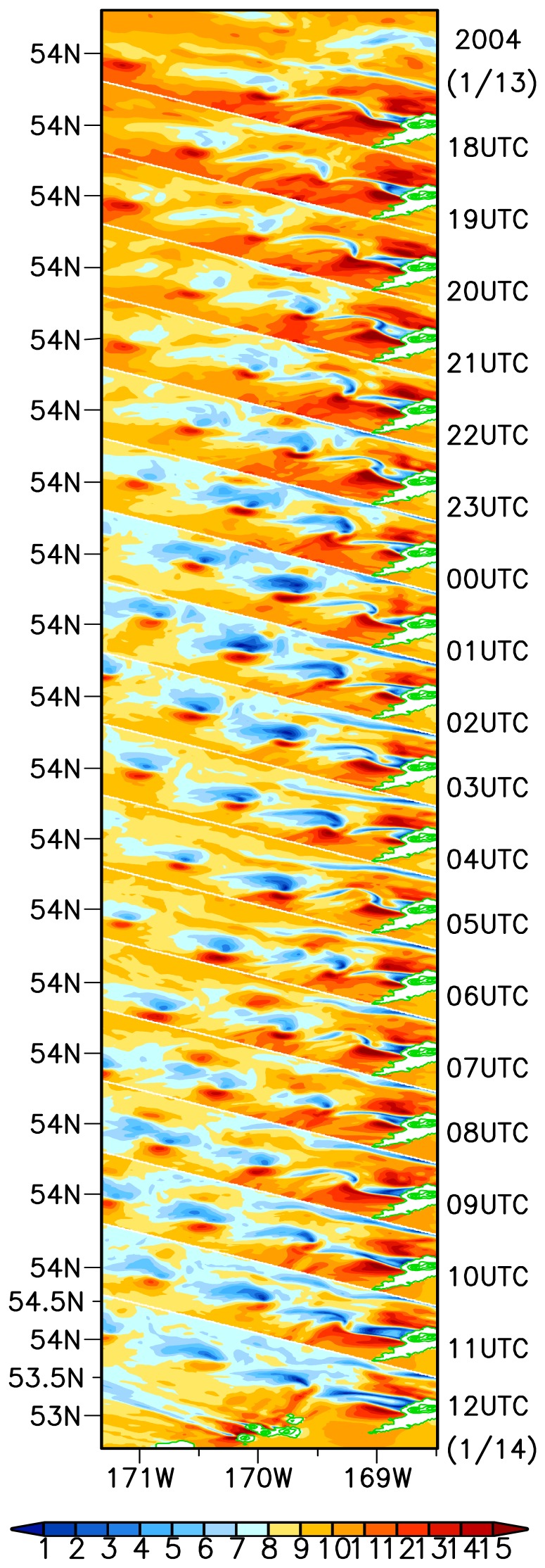
Reconstruction of the time evolution of the AVS during a period from 1800 UTC 13 to 1200 UTC 14 January 14, 2004. Colors and lines are the same as Figure 2a.

To understand the temporal evolution of this individual vortex street, we reconstruct the hourly wind field at 10 m above the sea surface along the vortex street between 1800 UTC 13 and 1200 UTC 14 January 2004 in Figure 4.

At early stage, the wind pattern develops an oscillatory-like wave downwind of the mountain and the weak wind region is not obvious. At 2000 UTC, the oscillation wake becomes so strong that a series of vortices break away from the mountain and move northwestward. The vortex street becomes the most distinguished during the period from 0100 UTC to 0300 UTC, and then gradually weakens.

Thomson et al. (1977) suggested the undisturbed wind velocity or free upstream speed *U_0_* and the effective diameter *d* for the barrier are determined at the 750 m level. We note that the upstream wind at the 750 m level almost remains unchanged, being 10-12 m s^-1^ in the earlier period of simulation (from 0000 UTC to 1100 UTC 13 January 2004) and 9-11 m s^-1^ in the latter period of simulation (from 1100 UTC 13 to 1200 UTC 14 January 2004). Table 1 gives characteristic values for the vortex street of Umnak Island, calculated from the model simulation. From the appearance of the first vortex at 2000 UTC 13 January, there are a total of 8 other vortices shedding off the mountain until 1100 UTC 14 January. Thus the average shedding rate is estimated to be 1 hour and 50 minutes. This result is shorter than that (2hr 48 minutes) from Thomson [1977], but longer than that (35 minutes or 48 minutes) from Li *et al.* [2000]. The average translation speed of the vortices is measured at about 8 m s^-1^. Other characteristic values for the vortex street of the Umnak Island are given in Table 1. These values are in the same range as previous studies.

## 5. Discussion and Conclusions

**Figure 5.**
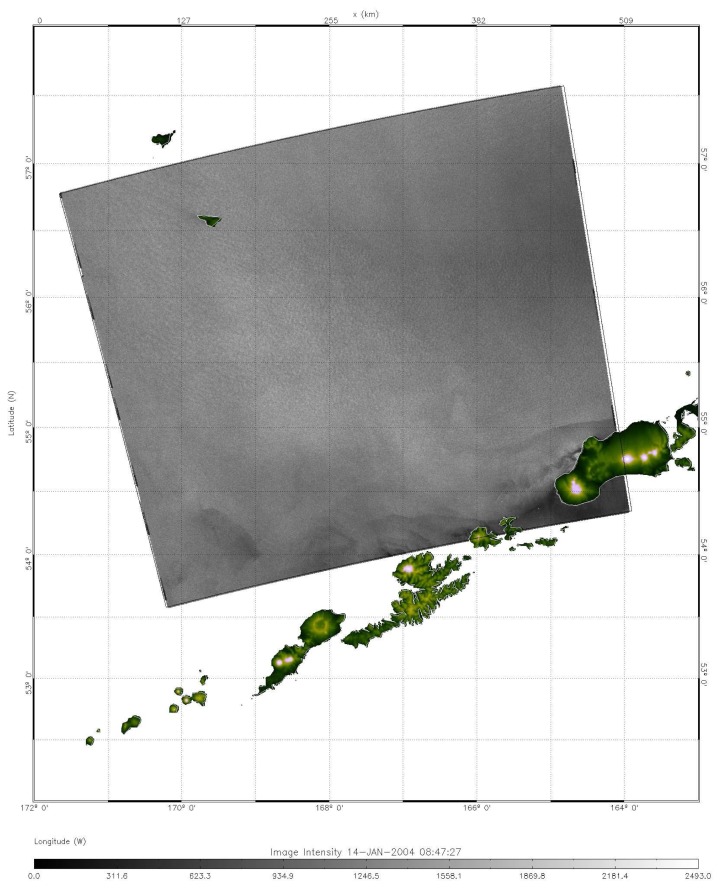
An ENVISAT ASAR image taken at 22:45:01 on January 13, 2004, showing no obvious AVS pattern. This indicates that the life span of AVS in this case study is shorter than one day.

The life span of the AVS depends upon the low-level atmospheric circulation. An ENVISAT Advanced SAR image taken at about 4 hours after the second RADARSAT SAR image (Figure 1b) is presented in Figure 5. One can see that the AVS is significantly weakened. There is no obvious vortex structure, but the low wind speed area are still exist near 54°N, -168°W area. This indicates that the AVS lasts shorter than one day.

**Figure 6.**
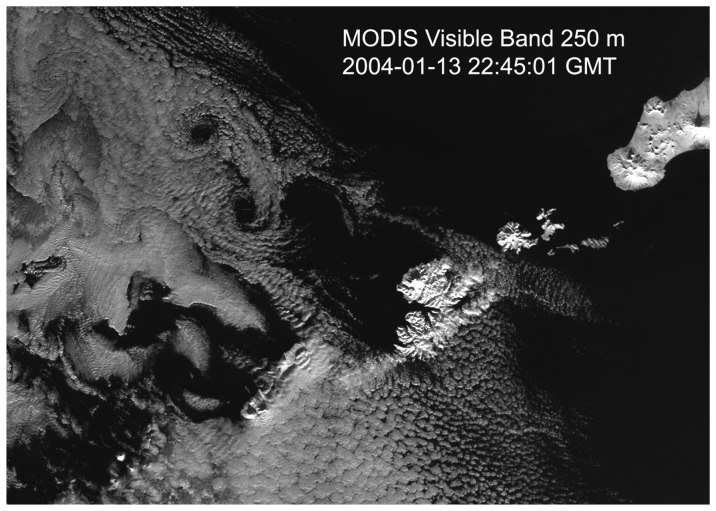
A full resolution (250 m) MODIS cloud visible band image taken between 2 SAR Images (2004-01-13 22:45:01) showing the AVS cloud pattern.

As we discussed in the introduction part, this low-level atmospheric circulation can also modify the cloud structure when the atmospheric moisture content is high. Figure 6 is a visible band image from Moderate Resolution Imaging Spectroradiometer (MODIS) instrument onboard NASA/EOS Terra satellite. The spatial resolution of this visible image is 250 m. The image was taken at 22:45:01 on January 13, 2004. The MODIS imaging time is between the acquisition times of the two RADARSAT SAR images in Figure 1. A pair of AVS can be clearly seen on the lee side of Umank Island on this MODIS image. This indicates the AVS happens in the entire MABL. The characteristics of SAR and MODIS images are given in Table 2.

AVS is one of the most significant mesoscale-β phenomena in marine atmospheric boundary layer. It has been detected on satellite cloud images for more than four decades. The recent SAR measurements not only show the AVS spatial structure in a much higher resolution than that of weather satellite (both polar and geostationary) but also can quantitatively provide the surface wind field. In this study, the SAR-derived sea surface wind speed is estimated with CMOD5 model modified for HH polarization. The wind direction input is from the Navy NOGAPS model. To understand the full cycle of the AVS generation and evolution, we implement a high-resolution numerical weather model MM5 with actual meteorological conditions as inputs to simulate the actual near surface wind field when the SAR images were taken. The surface wind fields from the RADARSAT-1 SAR measurements and that simulated by MM5 model is in good agreement.

The basic characteristics of the AVS are obtained through the model simulation, and compared with the previous observations. This case study suggests that combination of MM5 model and SAR image have significant implications in understanding the MABL phenomena near the coastal areas.

## Figures and Tables

**Table 1.** Characteristic values for the vortex street of the Umnak Island. *H* refers to the predominant mountain peak with *d* its effective width at the 750 m level. *U_0_* is the undisturbed wind velocity; *U_e_* the propagation speed of the vortex; T the period of the vortex formation and f the vortex shedding rate or frequency of formation; *S* the Strouhal number; *R_e_* the Reynolds number and υ the kinematic viscosity.

Topographic region	*H* (m)	*d* (m)	*U_0_* (m s^-1^)	*U_e_* (m s^-1^)	*T*=*1*/*f*	*S*	*R_e_*	*υ* (m^2^ s^-1^)
Mount Vsevidof (Umnak Island)	1850	13	11	8	1h50min	0.179	72-179	799-1986

**Table 2.** The imaging time characteristics of SAR and MODIS images used in this study.

Imaging Time (UTC)	Satellite	Instrument	Spatial resolution (m)
01/13/2004 17:31:00	RADARSAT-1	SAR ScanSAR	100
01/13/2004 22:45:01	EOS/TERRA	MODIS	250
01/14/2004 04:56:34	RADARSAT-1	SAR ScanSAR	100
01/14/2004 08:47:27	ENVISAT	ASAR ScanSAR	75
